# Hemorrhoids, Fissures and Fistula in Ano, and Their Treatment

**Published:** 1888-09

**Authors:** T. J. Bennett

**Affiliations:** Austin, Texas


### HEMORRHOIDS, FISSURES AND FISTULA IN ANO, AND THEIR TREATMENT.

BY T. J. BENNETT, M. D., AUSTIN, TEXAS.

Read at Austin District Medical Society, June 13, 1888.

I HAVE been somewhat at a loss to know how I could present the three subjects of this paper so as to get the main points before the Society and yet remain within due limits as to the time allotted for papers to be read.

The chief object of a paper, if I am properly informed, is to elicit discussion, thereby obtaining the views of many, instead of one. Discussing first the subject of

HEMORRHOIDS,

I may state that about the only grounds for difference of opinion is in the treatment. Just how to cure our patients with the least possible outlay of risk, time and pain is certainly the great *desideratum.*

The pathology and etiology of hemorrhoids need hardly be referred to here. But little advance in these respects has been made during the past half century.

The classification has been long settled as follows: relative to the sphincter ani muscle, hemorrhoids are internal and external. These are known as simple varicosities of the capillaries of the hemorrhoidal veins and arteries, and are situated immediately beneath the mucous membrane of the anus.

In order to refresh the memory, in passing, I may state that the vessels from which external and internal hemorrhoids are formed, are of two systems or sets, and for the most part are entirely distinct. In short, external hemorrhoids are derived from the external and middle hemorrhoidal veins which convey the blood to the heart through the general venous system; that is, through the ileac, and ascending vena cova. While the internal variety is formed from the internal hemorrhoidal vein, which carries the blood to the heart through the portal system, passing through the liver.

It is true, the plexiform condition of the anal and rectal vessels, sometimes anastomose freely, making it quite impossible to differentiate in all cases as to which system a given tumor might belong, but from an etiological standpoint the tumors may be wholy distinct. If this view be correct, there is a practical value in the differentiation. For instance, an internal hemorrhoid may be secondary to a more important pathological lesion, such as a congested liver, rectal tumor or stricture, and, if treated as a primary affection, like the external hemorrhoid, it would be to the neglect of the more important disease. If a protruding tumor will return upon gentle, continuous pressure, it may be regarded as originating from the rectal capillaries of the portal system. This is known as Allingham's device for determining whether a given hemorrhoid belongs to the external or internal variety.

It sometimes occurs that an internal hemorrhoid, by repeated straining at stool and by spasmodic action of the sphincter, is forced outside, and may become, in fact, an external hemorrhoid, but such a condition can take place only when there exists a free anastomosis which usually ceases afterwards.

As to the causation of hemorrhoids, it may be stated in general terms, that anything is a cause which obstructs the free return of blood through the hemorrhoidal veins. It is to be remembered that the veins in this region, including the entire portal system, are without valves, and are liable to over-distention from any of the numerous causes—such as over-loaded colon, pelvic tumors, uterine displacements, congested liver, etc. Perhaps the most frequent of the exciting causes is constipation—the irregularity of emptying the bowels, especially during pregnancy, and the consequent straining in order to pass hard fecal masses. The erect posture, acting by gravitation, has a great deal to do with the production of these tumors, for it is a well known fact that the lower animals are not thus afflicted, due, in part, no doubt, that the rectum lies in a plane horizontal to the body, instead of vertical, as in man.

This disease is too common, and your time too precious, for me to detail the symptoms. I would call attention, however, to one symptom, which I think is worthy of investigation and remembrance. I refer to pain in the hip, which is sometimes present, and is liable to lead the physician to a diagnosis of rheumatism or sciatica. This is especially true when the rectal pains are not very severe, and there is no hemorrhage to attract attention to the bowel. An examination, however, will frequently reveal a small hemorrhoid, which, if removed, relieves the pain in the hip, it being only referred. Two such cases have come under my observation.

### TREATMENT.

This may be palliative or radical. If the tumors are protruding and painful, I usually employ soothing applications of warmth or cold, and gentle pressure with the fingers. This will often give temporary relief, and cause the hemorrhoids to return within the bowel. Cocaine, in the form of lanoline ointment, I have found to be an admirable application to relieve pain and check hemorrhage. Then keeping the bowels open with the fluid extract of cascara segrada, two or three drops taken at meal times, in coffee, to disguise the disagreeable taste of the drug, and adopting regular times for going to stool, will do about all that may reasonably be expected from palliative treatment.

The curative, or radical, treatment, consists in a number of different procedures, chief among which are the well-known operations by the ligature, the clamp and cautery, and the injection of carbolic acid. Divulsion of the sphincter is now generally employed previous to operation by either of the above methods, excepting that of the injection of carbolic acid.

It is a well known fact that all the smaller tumors get well without further interference after the muscle is thoroughly paralyzed.

The plan which I prefer and adopt exclusively, is somewhat different from any of the above. It consists in first paralyzing the sphincter by placing it upon the stretch in the usual way, and then seizing the tumors with an ordinary pair of dressing forceps and breaking them down roughly with a meta-carpal saw, or, more frequently, with the point of a scalpel. The aim is to destroy the hemorrhoid by making a contused or lacerated wound, so there will be no hemorrhage. Inordinate bleeding has never followed any operation by this method. Even the arterio-venous variety of hemorrhoids of the largest size do not bleed excessively. Not more than an ounce or two of blood ever escapes during any operation.

I do not see this operation referred to in any of the literature at hand, yet I do not doubt but it is more or less familiar to the profession. To be sure, the principle is a correct one and an old one, viz: that a contused or lacerated wound does not bleed like an incised wound. To all whom I have suggested this operation, the fear of hemorrhage seems to be the main objection. This apprehension, I may remark, will be readily dispelled upon the adoption of the plan in a few cases.

The patient is usually well in eight or ten days, in fact he is up and about by the time he has regained sufficient strength in the sphincter to prevent involuntary stools upon assuming the erect position.

I prefer this operation to the ligature, for the following reasons:

1. Because it is quicker and easier performed.

2. Because there is no ligature to slough off, and no constricted tissue to cause pain for two or three days after the operation.

3. Because the wound which is made by breaking down the tumor is well before the ligature could slough off.

4. Because there is no ulcer remaining, as in the method by the ligature, to require eight or ten days more to heal.

I prefer it to the clamp and cautery, because it is less trouble to perform, and never leaves contracting cicatrices behind.

I prefer it to the plan of injection of carbolic acid, because it is more certain and one operation suffices. If an operation must be performed, and an anæsthetic given, the treatment employed should be both certain and safe. The carbolic acid method is neither certain nor safe, although an anæsthetic is not often required other than cocaine. The most violent proctitis and paraproctitis I ever witnessed, resulting in abscess, was produced by a 30 per cent. aqueous solution of carbolic acid injected into an internal hemorrhoid.

I usually remove jags and external hemorrhoids by simple excision under cocaine and then employ a continuous suture of catgut to close the wound and check the hemorrhage.

In concluding what I have to say on the subject of the treatment of hemorrhoids, I would state that I do not wish to be understood as condemning the time-honored ligature, the clamp and cautery and the carbolic acid treatment as procedures unworthy of confidence and employment, but I have sought to bring out the operation which I adopt, by contrasting it with the objectionable features of the other methods.

The thing to do is plain, and any of the operations mentioned, if properly carried out, will be successful. There is, perhaps, no disease which yields such universal good results from surgical operation as hemorrhoids.

I adopted the method of breaking down the tumors as described above (which, by the way, is an entirely different procedure to "crushing") early in practice, and I have found the plan an admirable one, and I commend it to the profession.

FISSURES.

Fissures may be defined as cracks in the mucous membrane surrounding the anus and within the grasp of the sphincters.

They are in a line with the bowel, and may penetrate only partially through the mucous membrane or may extend down to the sphincter muscle, presenting the appearance of deep ulcers. Fissures rarely extend more than an inch in length. They are usually produced by the forcing of hard fecal masses through the bowel at a time when the surrounding parts are inflamed from hemorrhoids or a surgical operation. Foreign bodies, such as pins, pieces of bone, etc., swallowed and lodged at the anus, may cause fissures or ulcers.

One of the most important points connected with the subject is the clear differentiation between the simple, syphilitic and malignant forms of the disease. Without entering into detail, I will state briefly how I am in the habit of determining to which class a given fissure or ulcer may belong. If there is no history of syphilis and no direct physical signs, no persistently offensive discharges, no glandular enlargements, and no special induration about the base of the ulcer, the slit being plain and simple, I am very certain to pronounce the lesion a simple fissure ; on the other hand, if there be a history of syphilis, with or without constitutional signs, other than the rectal lesion, the ulcer being soft or but slightly tinctured and persistent, I diagnose the breach to be syphilitic. If the base should be rather hard, discharge offensive and profuse, the inguianl glands enlarged and tender, no history of syphilis, the patient cachectic, the lesion is most likely malignant. The pain is usually quite severe, whether the ulcer be simple, syphilitic or malignant, and differs from the pain in hemorrhoids and fistulæ by being more sharp and cutting in character.

### TREATMENT.

After the diagnosis has been made, and the cause (should it be a foreign body) removed, the treatment of a simple fissure is itself simple. Perfect cleanliness by the use of antiseptic washes from time to time, and occasionally, when the sore is indolent, a solution of nitrate of silver, one or two drachms to the ounce of water, should be applied at intervals of three or four days. I have found these means to succeed in the majority of simple cases, yet, where the case is obstinate, of long standing, and the general health reduced, constitutional treatment renders valuable aid. But in all cases in which milder means have been employed and failed, divulsion of the sphincter becomes necessary to success. Divulsion not only renders the sore more accessible, but it greatly facilitates cleanliness, and acts directly curative by relieving the tension, which is an aggravating cause of the fissure. In my opinion, simple excision, and stitching the wound together with cat gut, preferably, or silk, is the best operative procedure extant.

The syphilitic fissure, or ulcer, is subject to about the same treatment as the simple, with the addition, always, of constitutional medication.

The malignant form, I have always treated palliatively, yet, if taken in time, extirpation offers some hope of relief, temporarily at least.

### FISTULA IN ANO.

This disease is next in frequency to hemorrhoids, and it is quite as painful and persistent.

Properly speaking, "a common fistula is a sinus leading from within the rectum to an opening on the skin more or less remote from the anus."

Fistulæ are classed as external blind, and internal blind, respectively, as the external or internal sinus is opened or closed.

They are the result of abscesses in the cellular tissue which so abounds about the rectum. The causes which lead to abscess and fistula are many, prominent among which are bruises, hemorrhoids, fecal impactions, foreign bodies, etc. When once established, the fistulous opening seldom heals spontaneously. This is due, I think, to the spasmodic action of the sphincter, which is maintained and increased by the stimulus of inflammation, which cannot subside of itself while the muscle thus acts. Patients suffering from fistula in ano lose more in general health, as a rule, than those suffering from hemorrhoids or fissures. The operative measures for the cure of fistula are quite as satisfactory as for the other two diseases.

The operation most frequently employed by the regular profession, as you are well aware, is that of dividing the sphincter, cutting all holes into one, as it were, and then treating the wound antiseptically with washes and ointments. The object is to secure drainage and cleanliness.

The horse hair method of Hippocrates is not much in vogue now, nor is the elastic ligature.

What is known as the "itinerant," because the idea originated among the "traveling doctors," is now growing rapidly into favor, after having been modified and systematized by Dr. Matthews, of Louisville.

The original plan was simply to probe the sinus and inject it with a solution of peroxide of hydrogen.

Dr. Matthews added the laminaria tent for dilating the external opening.

I am in the habit of simply paralyzing the sphincter muscle, and using, as required, nitrate of silver washes, say one drachm to the ounce of water. This solution is antiseptic, and stimulating to the indolent character of the ulceration. Some fistulous openings need no further treatment than the mere removing of the tension by the muscle. Drainage and cleanliness are thus secured without having a large wound to heal by the slow process of granulation, as in the cutting treatment.

There is no denying the fact that the dividing of the sphincter is a very successful surgical procedure, but divulsion virtually accomplishes the same thing, and requires much less time of the patient.

I have never found it necessary to open the sinus into the bowel in cases of internal blind fistula, but the external blind fistula is usually more or less filled with pus or fecal matter, and should be let out.

Patients suffering from tuberculosis, or other wasting disease, are frequently the victims of fistula. It is over this class of patients the physician is most troubled, as to how, when, or whether an operation should be performed at all.

The "seton" idea of cure, strange to say, is still to be met with once in a while. We are gravely informed that to check an issue here will "throw the disease back upon the lungs," and death will be the result. To be sure, good judgment and common sense are always at a premium. It is especially so in medicine, and doubly so in the department of surgery. If the patient is too far advanced to undergo an operation, it would be criminal to knowingly kill him by an operation. But the fact should never be lost sight of that two issues are greater than one, and that a physician's solemn duty is to do the best he can under the circumstances.

